# The Chaperone-Like Activity of α-Synuclein Attenuates Aggregation of Its Alternatively Spliced Isoform, 112-Synuclein *In Vitro*: Plausible Cross-Talk between Isoforms in Protein Aggregation

**DOI:** 10.1371/journal.pone.0098657

**Published:** 2014-06-03

**Authors:** Krishna Madhuri Manda, Deepthi Yedlapudi, Srikanth Korukonda, Sreedhar Bojja, Shasi V. Kalivendi

**Affiliations:** 1 Centre for Academy of Scientific & Innovative Research, CSIR-Indian Institute of Chemical Technology (CSIR-IICT), Hyderabad, Andhra Pradesh, India; 2 Centre for Chemical Biology, CSIR-Indian Institute of Chemical Technology (CSIR-IICT), Hyderabad, Andhra Pradesh, India; 3 Inorganic and Physical Chemistry Division, CSIR-Indian Institute of Chemical Technology (CSIR-IICT), Hyderabad, Andhra Pradesh, India; Hertie Institute for Clinical Brain Research and German Center for Neurodegenerative Diseases, Germany

## Abstract

Abnormal oligomerization and aggregation of α-synuclein (α-syn/WT-syn) has been shown to be a precipitating factor in the pathophysiology of Parkinson's disease (PD). Earlier observations on the induced-alternative splicing of α-syn by Parkinsonism mimetics as well as identification of region specific abnormalities in the transcript levels of 112-synclein (112-syn) in diseased subjects underscores the role of 112-syn in the pathophysiology of PD. In the present study, we sought to identify the aggregation potential of 112-syn in the presence or absence of WT-syn to predict its plausible role in protein aggregation events. Results demonstrate that unlike WT-syn, lack of 28 aa in the C-terminus results in the loss of chaperone-like activity with a concomitant gain in vulnerability to heat-induced aggregation and time-dependent fibrillation. The effects were dose and time-dependent and a significant aggregation of 112-syn was evident at as low as 45°C following 10 min of incubation. The heat-induced aggregates were found to be ill-defined structures and weakly positive towards Thioflavin-T (ThT) staining as compared to clearly distinguishable ThT positive extended fibrils resulting upon 24 h of incubation at 37°C. Further, the chaperone-like activity of WT-syn significantly attenuated heat-induced aggregation of 112-syn in a dose and time-dependent manner. On contrary, WT-syn synergistically enhanced fibrillation of 112-syn. Overall, the present findings highlight a plausible cross-talk between isoforms of α-syn and the relative abundance of these isoforms may dictate the nature and fate of protein aggregates.

## Introduction

Parkinson's disease (PD) is a progressive neurodegenerative disorder associated with the loss of dopaminergic (DA) neurons in the substantia nigra (SN) pars compacta [1], [2]. α-Synuclein (α-syn), a pre-synaptic protein is known to play a crucial role in the pathophysiology of PD [3]–[6]. Also, α-syn was found to be the major constituent of Lewy body (LB) aggregates, the intracellular proteinaceous inclusions in PD which are considered as pathological hallmarks [7]. The gene structure of α-syn reveals the presence of 6 exons and so far at least three alternatively spliced transcripts were identified [8]–[10]. α-Syn is a 140-amino acid residue protein consisting of three domains: the N-terminal domain (amino acids 1–60) with five amphipathic helices interacting with lipid membranes, the central hydrophobic NAC domain (amino acids 61–95) and a highly charged acidic C-terminal domain (amino acids 96–140) conferring the negative charge to the protein [11]–[14]. Recently, it was shown that a decrease in the C-terminal charge favors the formation of fibrillar species shifting the equilibrium [15]. *In vitro*, α-syn aggregates and forms fibrils with similar morphology and properties to that of the amyloid fibrils extracted from Lewy bodies [16], [17]. The initial observation that *A30P* α-syn may have a tendency to accumulate as oligomers instead of mature fibrils led to the suggestion that α-syn may have a similar toxic mechanism [18], [19]. The small oligomers of α-syn are found to be more toxic when compared to the mature fibrils [20]. It has been suggested that the long range interactions between the NAC and C-terminal domain inhibit the formation of mature fibrils maintaining the stable natively unfolded structure of α-syn [21], [22]. Deletion of the C-terminus has been proposed to enhance the aggregation of α-syn and the C-terminal truncated forms were found to be more prone for fibrillation than the full length protein [23]–[26]. It is now well recognized that the abnormal oligomerization and aggregation of α-syn play a pivotal role in the Lewy body formation and the pathogenesis of Lewy body diseases [27].

Recent discoveries highlight that alternative splicing of α-syn may also play a crucial role in the mechanisms mediating PD [28]–[31]. So far, deletion of exon 3 [126-Syn] or exon 5 [112-Syn] or both exon 3 and 5 [98-Syn] have been identified [32], [33]. The aggregation propensities of these isoforms have been proposed based on the net charge of the protein as compared to wild-type synuclein (WT-syn) [34]. Our earlier study identified an induced-alternative splicing of α-syn by various Parkinsonian mimetics resulting in the generation of 112-syn (112-synuclein) and overexpression of 112-syn was found to be deleterious to dopaminergic cells [35]. Further, region specific transcript abnormalities were also noticed in patients as well as transgenic mouse models of α-synucleinopathies [36], [37]. Hence, in the present study we sought to understand the biological consequences arising out of the increased generation of 112-syn and its functional implications in the presence or absence of WT-syn to understand whether the relative abundance of α-syn isoforms play a crucial role in the events leading to protein aggregation.

## Materials and Methods

### Materials

Isopropyl-β-D-thiogalactopyranoside (IPTG), Glutathione-Sepharose 4B, Thrombin from bovine plasma, Aldolase (ALD) from rabbit muscle, ThioflavinT (ThT) and all other chemicals were purchased from Sigma, St. Louis, MO, USA. Restriction enzymes were obtained from Fermentas Inc, Fisher Scientific, PA, USA. All other reagents were of analytical grade.

### Methods

#### Construction of WT and 112-syn expression plasmids

Human SH-SY5Y dopaminergic cells were treated with 2 mM MPP^+^ for 24 h, for the induction of 112-syn as described previously [35]. Following the termination of the experiment, the medium was aspirated and 1 ml of TRIzol reagent (Invitrogen Inc, USA) was added to the cells in 6-well plates and total RNA was extracted using the manufacturer's protocol. 5 µg of RNA was used for the first strand cDNA synthesis using a first strand cDNA synthesis kit (Thermo Scientific, USA) according to the manufacturer's protocol. Full-length WT-syn and 112-syn mRNA were amplified using high-fidelity PCR supermix (Hi FI PCR supermix, Invitrogen Inc, USA) using the forward (5′-AA***CCCGGG***CATGGATGTATTCATGAA AGGACTTTCA-3′) and reverse (5′-AA***CTCGAG***AGATATTTCTTAGGCTTCAGGTT CTAGT-3′) primers containing *SmaI* and *Xho I* restriction sites (shown in bold). Following PCR, the amplified product was digested with *SmaI* and *Xho I* and ligated into pGEX-4T-1 plasmid (GE Healthcare Life Sciences, USA) that was predigested with the same restriction enzymes in order to obtain the plasmids expressing the GST (Glutathione-S-transferase) fusion constructs of WT-syn and 112-syn respectively.

#### Expression and purification of α-syn isoforms

The GST-synuclein fusion constructs were transformed into BL21 (DE3) pLysS strain of *Escherichia coli* and protein expression was induced with 1 mM IPTG. The recombinant GST-synuclein fusion proteins of WT and 112-syn were purified by affinity chromatography using glutathione sepharose-4B beads as described previously [38]. The fusion proteins were subjected to thrombin cleavage and subsequently mixed with glutathione sepharose-4B beads and p-aminobenzamidine agarose to trap any cleaved-off GST and thrombin. The proteins were concentrated using Centricon concentrators (Millipore, USA) of molecular mass cut-off 10 kDa and further purified using Superdex75 (GE Healthcare) gel-filtration column equilibrated with phosphate-buffered saline, pH 7.4. The protein concentrations were determined by bicinchoninic acid (BCA) protein assay kit [Thermo Fisher Scientific, IL, USA] using bovine serum albumin as a standard and the purity of the proteins was verified by SDS-PAGE. Protein samples were centrifuged at 12,000×*g*, for 30 min at 4°C prior to the experiments to remove any possible particulates.

#### Measurement of chaperone like activity of α-syn

The ability of WT-syn and 112-syn in suppressing the heat-induced aggregation of substrate proteins like aldolase was monitored as described previously with slight modifications [39], [40]. Briefly, individual protein solutions of aldolase (0.1 mg/mL), WT and 112-syn, or mixtures of both WT and 112-syn with aldolase at varying concentrations were prepared in phosphate buffered saline (PBS) pH 7.4 and heated at 65°C for a period of 10 min in a quartz cuvette. The light scattering of the solution was monitored at 360 nm as a function of time using a thermostatted spectrophotometer (Jasco).

#### Turbidometric analysis of 112-syn

Turbidity measurement as an assay for protein aggregation was performed according to the established protocols [40]. Briefly, 112-syn at varying concentrations (0.2, 0.4, 0.6 mg/mL in a final volume of 100 µl) in phosphate buffered saline (PBS) pH 7.4 was incubated individually at three different temperatures of 45°C, 55°C, and 65°C for a period of 10 min in a quartz cuvette. The light scattering of the solution was monitored at 360 nm as a function of time using a spectrophotometer equipped with thermostatted cuvette holder (Jasco). In experiments where interdependency of isoforms was analyzed, 112-syn (0.4 mg/mL) was incubated with WT-syn at 1∶0.5, 1∶1 and 1∶2 ratios and kinetics of aggregation was analyzed by turbidometry.

#### Fibril formation

Solutions of both WT and 112-syn (0.5 mg/mL) in 20 mM Tris buffer, pH 7.5 were incubated under shaking conditions (1000 rpm) at 37°C in a thermo mixer (Eppendorf) as described previously [41]. Aliquots from the incubation mixtures were withdrawn at different time intervals for fluorescence measurements and transmission electron microscopy (TEM) analysis. In experiments where interdependency of isoforms was analyzed, WT-syn (0.25, 0.5 and 1.0 mg/mL) and 112-syn (0.5 mg/mL) were incubated at different ratios and kinetics of fibrillation was analyzed by Thioflavin (ThT) staining.

#### Analysis of fibril formation by ThT binding assay

Fibril formation was monitored with ThT fluorescence as described previously with slight modifications [42]. Briefly, aliquots of 5 µl from the incubation mixtures were withdrawn at various time points, diluted to 100 µl with 25 µM ThT in 50 mM Tris buffer (pH 8.0). The fluorescence emission spectrum (470–600 nm) of the samples was recorded following the excitation of samples at 450 nm in an Enspire 800 model Perkin-Elmer multimode reader. The blank measurement recorded prior to the addition of proteins was subtracted from the signal obtained from each sample.

#### Transmission Electron Microscopy

An aliquot of 5 µl was withdrawn from the incubation mixtures at different time intervals and adsorbed onto a glow-discharged carbon-coated copper Formvar EM grid. After 1–2 min of adsorption, the grids were rinsed for 1 min with a few drops of 1% uranyl acetate, blotted with filter paper and air dried as described earlier [42]. The images were recorded with a TECNAI FE12 TEM (Eindhoven, The Netherlands) instrument operating at 120 kV. The photomicrographs were analyzed with SIS imaging software.

## Results

### Increased propensity of 112-syn to heat-induced aggregation is associated with the lack of its chaperone activity

**Figure 1:**
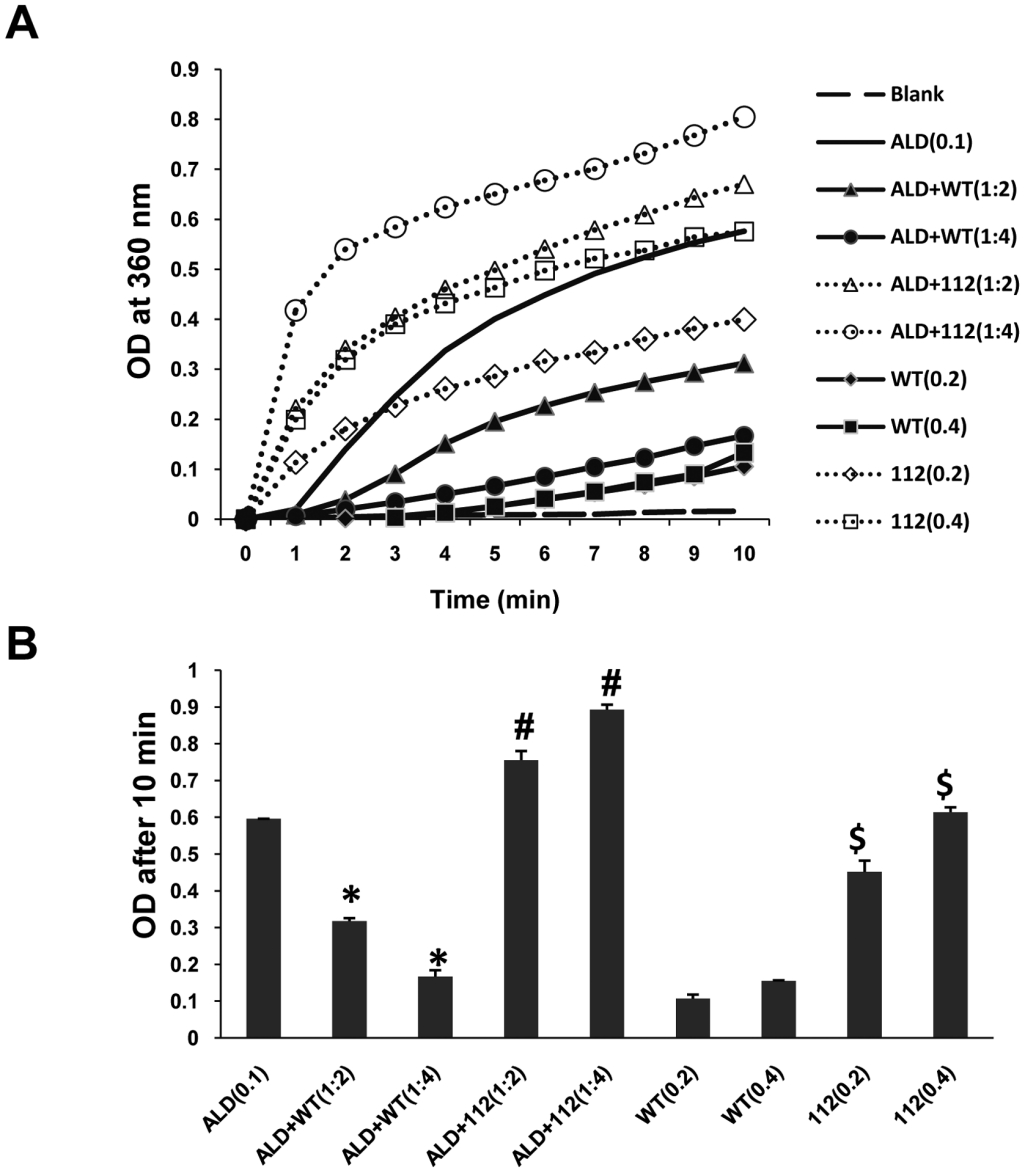
Effect of WT and 112-syn on heat induced aggregation of aldolase (ALD). (**A**) *In vitro* protein aggregation assay employing aldolase (0.1 mg/mL in PBS) was performed at 65°C in the absence or presence of WT and 112-syn at different ratios (1∶2 and 1∶4) and the light scattering was monitored at 360 nm for a period of 10 min. (**B**) The net change in the absorbance of reaction mixtures of (A) before and after 10 min of incubation at 65°C. Data are the mean ± SD of three separate experiments. * indicates p<0.01 as compared to aldolase (decrease); #p<0.01 as compared to aldolase (increase) and $ p<0.01 as compared to the respective controls (before heating).

The ability of α-syn to suppress the aggregation of non-native conformations of substrate proteins like aldolase has been widely employed as a measure of its chaperone like activity *in vitro*. Previously, it was shown that chaperone-like activity of α-syn was lost due to the lack of its acidic C-terminus [40]. Concomitant with the earlier findings, our initial studies aimed at understanding the functional consequence of deleted exon-5 in 112-syn also noticed that unlike WT-syn, 112-syn did not demonstrate any inhibitory effect on the heat-induced aggregation of aldolase [Fig. 1 A and B]. Results indicate that incubation of aldolase alone (0.1 mg/mL) at 65°C induced its aggregation and the absorbance values reached from 0 to ∼0.6 during the incubation period of 10 min [Fig. 1A and B]. Moreover, WT-syn suppressed the aggregation of aldolase in a dose dependent manner [Fig. 1A and B]; which corroborates with the earlier observations [39]. However, the absorbance of incubation mixtures, wherein, a standard amount of aldolase (0.1 mg/mL) when incubated with increasing concentrations of 112-syn demonstrated a dose dependent increase in the absorbance at 360 nm which is significantly more than values exhibited by aldolase alone [Fig. 1A and B]. Under similar conditions, incubation mixture consisting of 112-syn alone exhibited a dose dependent increase in the absorbance at 360 nm and this phenomena was not observed with WT-syn [Fig. 1A and B]. The obtained results prompted us to study the heat-induced aggregation propensity of 112-syn.

**Figure 2:**
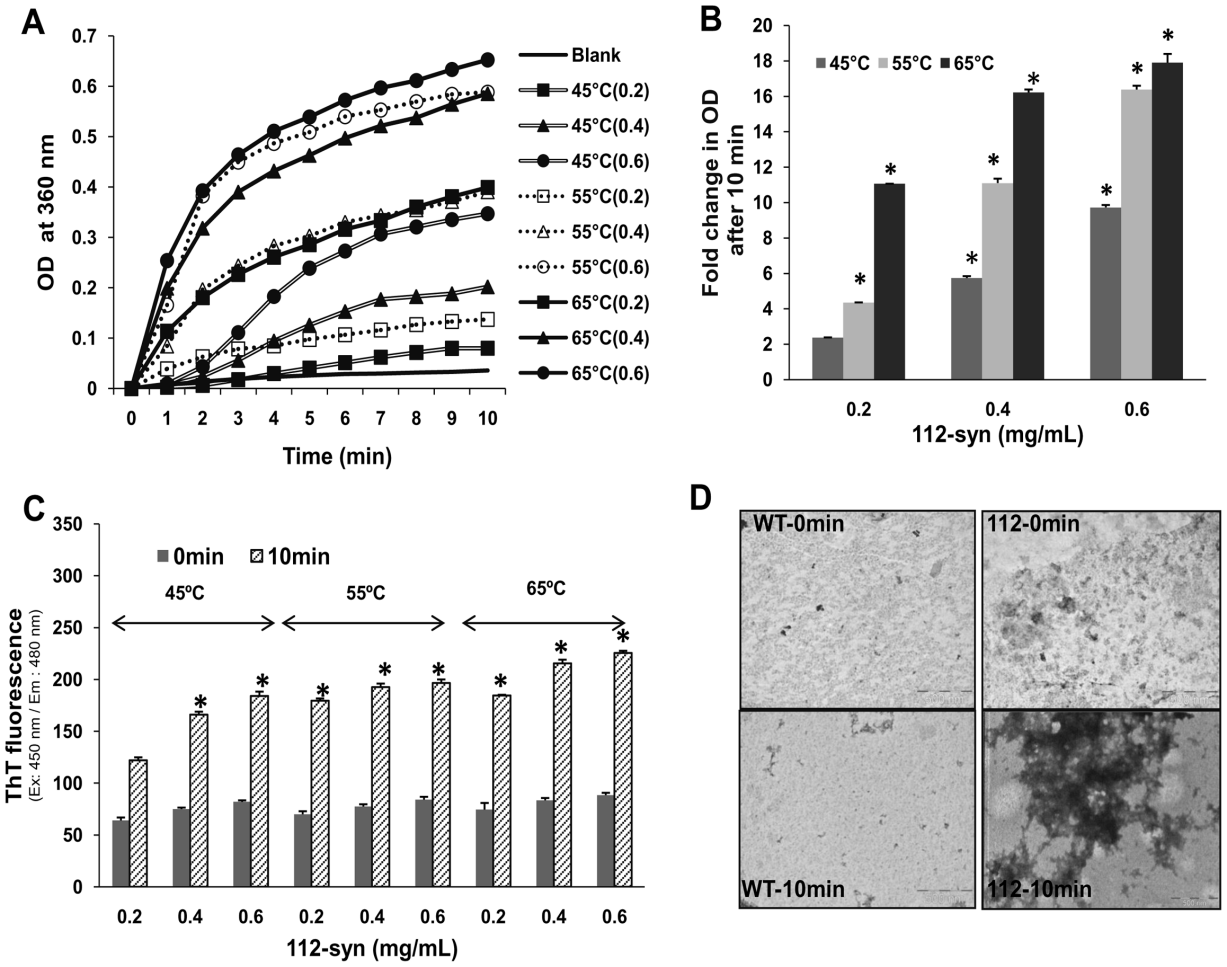
Temperature dependent aggregation of 112-syn. (**A**) 112-syn at different concentrations (0.2, 0.4, 0.6 mg/mL in PBS) was heated at 45°C, 55°C, and 65°C and the light scattering was monitored at 360 nm for a period of 10 min as described under “Methods” section. (**B**) Fold change in the net absorbance values from (A) at 360 nm before and after heating the reaction mixtures. (**C**) ThT fluorescence of reaction mixtures as indicated in (A) before (0 min) and after exposing the samples at indicated temperatures for 10 min.*p<0.01 as compared to 0 min readings for both (B) and (C). (**D**) Negatively stained TEM images of WT and 112-syn before and after exposure to 65°C for 10 min.

Next, we analyzed the kinetics of apparent light scattering of 112-syn (0.2, 0.4, 0.6 mg/mL) against heat-induced aggregation at three different temperatures (45, 55 and 65°C) over a period of 10 min. Results indicate a dose-dependent increase in the turbidity of 112-syn at all the temperatures examined [Fig. 2A and B]. While, 0.2 mg/mL of 112-syn displayed nearly 2-fold increase in the absorbance following 10 min of incubation at 45°C, the corresponding values for 0.4 and 0.6 mg/mL of protein were found to be 5 and 9 fold respectively. Similarly at 55°C, the three different concentrations of 112-syn (0.2, 0.4, 0.6 mg/mL) exhibited 4, 11 and 16 fold increase in the absorbance and at 65°C the fold increase in absorbance was found to be 11, 16 and 18 fold respectively [Fig. 2B]. The obtained data clearly demonstrate the enhanced vulnerability of 112-syn to variations in temperature and a significant increase in turbidity was evident at as low as 45°C. The binding of ThT to the crossed β-sheet structures (characteristic feature of amyloid fibrils of proteins) enhances its fluorescence and is being widely used to study protein fibrillation [43]. ThT staining of 112-syn before and after exposure to different temperatures though found significantly increased [Fig. 2C], but, the fold increase was not proportional to that of the observed turbidity under same experimental conditions (Fig. 2A and B). TEM images of 112-syn exposed to heat (65°C for 10 min) revealed the presence of ill-defined aggregates, however, under similar conditions no aggregates were found with WT-syn [Fig. 2D].

### WT-syn attenuates heat-induced aggregation of 112-syn

**Figure 3:**
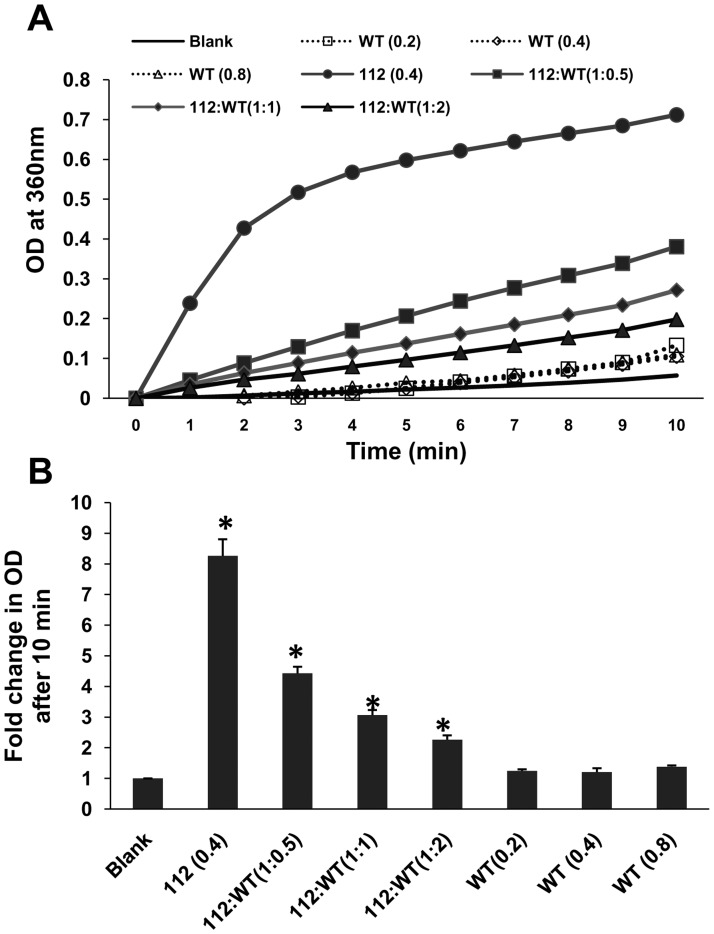
Effect of WT-syn on heat induced aggregation of 112-syn. (**A**) The chaperone-like activity of WT-syn was monitored by incubating 112-syn (0.4 mg/mL) in the presence or absence of different ratios of WT-syn as described in the “Methods” section. The time dependent increase in absorbance at 360 nm was recorded over a period of 10 min. (**B**) Fold change in the net absorbance values from (A) before and after 10 min of incubation at 65°C recorded at 360 nm.*p<0.01 as compared to 112-syn before heating.

Based on the above observations on distinctly different property of 112-syn as compared to WT-syn, we surmised whether the chaperone-like activity of WT-syn confers any protection towards the heat-induced aggregation of 112-syn. To examine this, we have exposed 112-syn to 65°C for 10 min in the presence or absence of WT-syn (in the ratios of 1∶0.5, 1∶1 and1∶2). Results demonstrate that WT-syn dose-dependently inhibited the heat-induced aggregation of 112-syn. At 1∶0.5, 1∶1 and 1∶2 ratios of 112-syn: WT-syn, the percent inhibition was found to be nearly 46, 62 and 72% respectively [Fig. 3A and B]. Under similar experimental conditions no gross changes in the absorbance were noticed in incubation mixtures consisting of WT-syn alone [Fig. 3B].

### Time-dependent fibrillation of 112-syn

**Figure 4:**
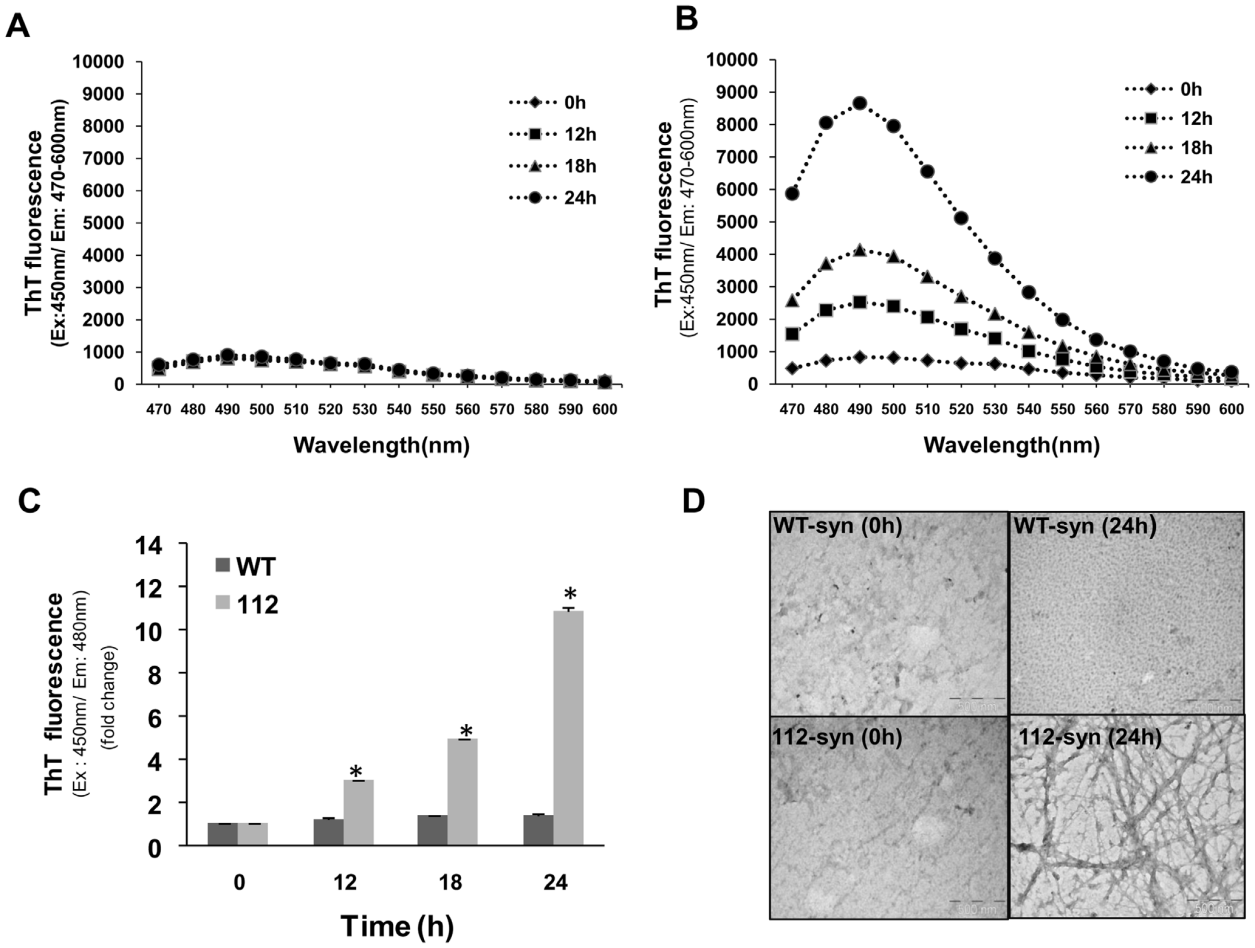
Time dependent fibrillation of WT and 112-syn. Time dependent fibrillation of WT-syn (**A**) and 112-syn (**B**) was monitored by the enhancement of ThT fluorescence. WT and 112-syn (0.5 mg/mL) at 37°C were incubated in 20 mM Tris buffer, pH 7.5 for indicated time intervals as described in the “Methods” section and the ThT fluorescence emission spectrum was recorded. (**C**) Fold change in the ThT fluorescence of WT and 112-syn incubated for different time intervals. Data presented are the mean ± SD of three separate experiments. (**D**) Aliquots from reaction mixtures incubated at 37°C for 24 h were adsorbed onto carbon coated grids and then stained with 1% uranyl acetate for 1–2 min. Negatively stained TEM images of WT and 112-syn are shown. *Scale bars*, 500 nm and data is a representative of three different fields of view. *p<0.01 as compared to 0 h readings for 112-syn.

Earlier reports demonstrated that α-syn aggregates in a time dependent manner forming large network of fibrils and a significant increase in ThT staining was reported following prolonged incubation (three days) at room temperature [42]. In order to understand the observed differences in ThT reactivity, we have incubated individually WT and 112-syn (0.5 mg/mL) under shaking conditions for 24 h at 37°C and subjected to ThT staining. The fluorescence emission spectra of ThT at different time points (0, 12, 18 & 24 h) for WT-syn and 112-syn are shown in Figure 4A and B. Results indicate that incubation mixtures containing WT-syn did not show any gross changes in the ThT emission spectrum during the initial 24 h time period [Fig. 4A]. Nevertheless, a significant increase in the aggregation of WT-syn was evident following prolonged incubation as reported in earlier studies (data not shown) [42]. Under similar incubation conditions, 112-syn exhibited a time dependent increase in the fibrillation based on the ThT emission spectrum [Fig. 4B]. Nearly, three, five and ten-fold-increase in ThT fluorescence of 112-syn was observed at 12, 18 and 24 h respectively, whereas, ThT fluorescence of WT-syn remained the same during the initial 24 h incubation [Fig. 4C]. TEM images of 112-syn under the above incubation conditions showed the presence of long extended network of fibrils and this effect was not observed with WT-syn [Fig. 4D], which is in coherence with the obtained ThT signal [Fig. 4A and B]. The data demonstrates that 112-syn possess enhanced vulnerability towards fibrillation as compared to WT-syn.

### WT-syn exacerbates fibrillation of 112-syn

**Figure 5:**
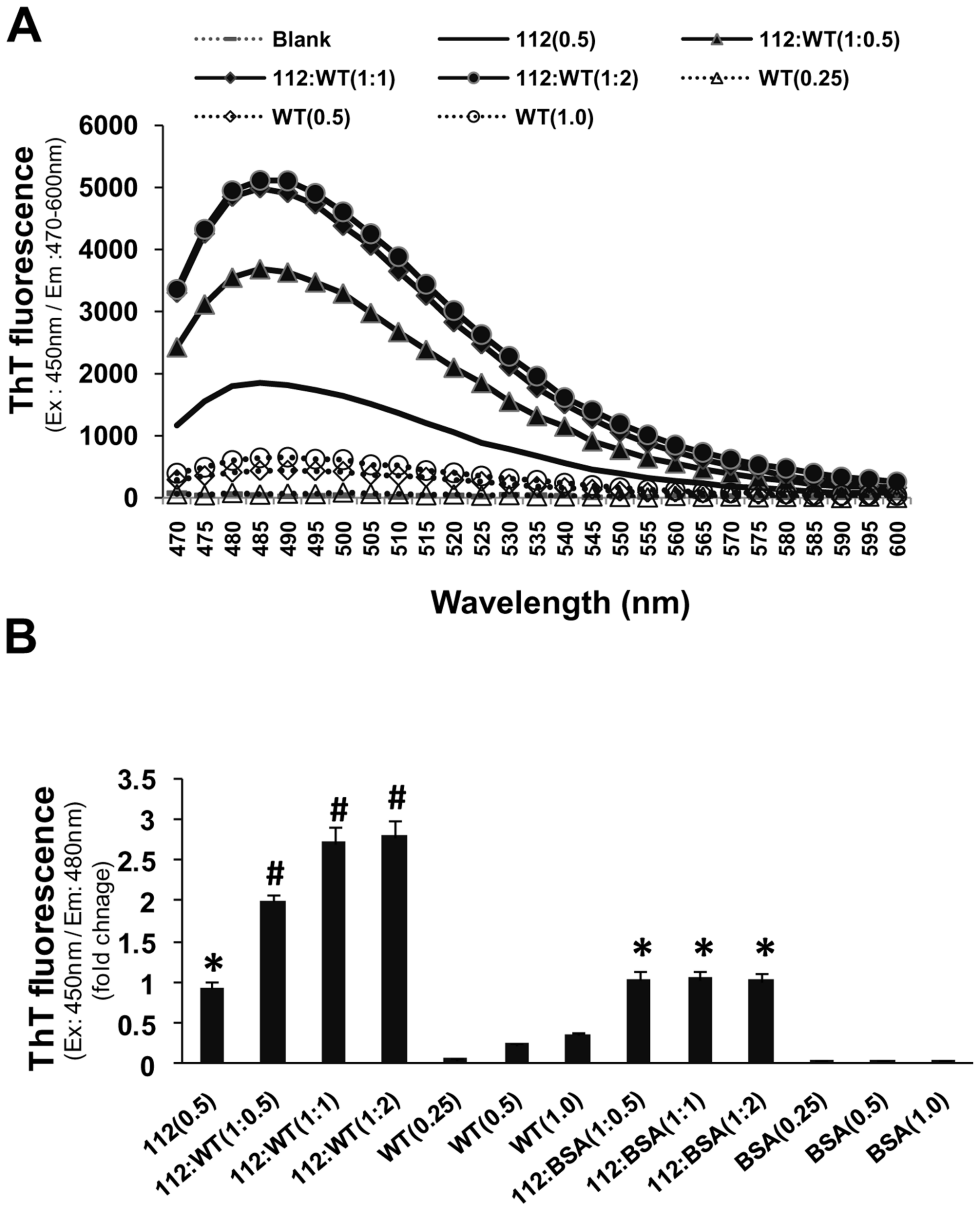
Effect of WT-syn on time dependent fibrillation of 112-syn. (**A**) 112-syn (0.5 mg/mL) was incubated in the presence or absence of different ratios of WT-syn at 37°C in 20 mM Tris buffer, pH 7.5 for 12 h. The increase in ThT fluorescence emission spectrum (470–600 nm) was recorded following excitation at 450 nm. (**B**) Fold change in the ThT fluorescence of 112-syn, incubated for 12 h in the presence or absence of different ratios of WT-syn. Data presented are the mean ± SD of three separate experiments. *p<0.01 as compared to 0 h reading for 112-syn; #p<0.01 as compared to112-syn alone.

Since, WT-syn was found to inhibit heat-induced aggregation of 112-syn, we next analyzed whether similar effects would also be evident on the fibrillation of 112-syn. To examine this, fibrillation of 112-syn was monitored by ThT staining following 12 h incubation at 37°C in reaction mixture containing 112-syn in the presence or absence of WT-syn at different ratios. The obtained data indicates that WT-syn dose-dependently increased the fibrillation of 112-syn as measured by ThT staining [Fig. 5A]. 112-syn when incubated with WT-syn at 1∶0.5 and 1∶1 ratio resulted in the enhancement of ThT fluorescence by nearly 1.9 and 2.7 fold respectively and the values remained nearly the same even at 1∶2 ratio [Fig. 5A and B]. A similar enhancement in ThT staining was not observed when 112-syn was incubated with different concentrations of bovine serum albumin, which rules out the possibility of differences in protein concentration between samples for the observed effects.

## Discussion

The presynaptic protein, α-syn is being considered as one of the precipitating factor in the pathophysiology of PD and gaining significance for its promising role in dementia with Lewy bodies (DLB) and related synucleinopathies [7], [44]–[45]. Earlier studies have identified the regulation of α-syn at various levels, such as, protein-protein interactions, post translational modifications effects on dopamine transporters, gene dosage or multiplication and alternative splicing [9], [46]. However, the events leading to the oligomerization and aggregation of α-syn were considered to be crucial in understanding the formation of LB aggregates, the hallmark of PD and related synucleinopathies [16], [17]. Amongst the proposed mechanisms, posttranslational modifications were shown to enhance the aggregation propensity of α-syn due to the formation of covalent links and stabilization of α-syn filaments [47]–[49]. Recent reports suggest that alternative splicing of α-syn is yet another regulation by which specific domains of proteins are deleted resulting in the generation of smaller isoforms with altered properties [33]–[35]. Also, PD-associated α-syn gene variants at the 3′ region influencing the expression of 112-syn and identification of region specific transcript abnormalities in related synucleinopathies emphasize the need to understand the role of these smaller isoforms in the pathophysiology of PD and related ailments [23]–[26] **** [36]–[37] **** [50].

Though the precise mechanism contributing to the generation of alternatively spliced isoforms of α-syn are not completely understood, our recent study identified the induced-alternative splicing of α-syn by Parkinsonian mimetics resulting in the generation of 112-syn. Also, overexpression of 112-syn was found to be deleterious to dopaminergic cells [35]. Based on these previous observations, the present study was conceived to understand the role of 112-syn by examining the behavior of this protein both *in vitro* and *in situ* in order to predict its plausible role in the events leading to protein aggregation/fibrillation.

The protein domain structure of α-syn reveals primarily three main regions, the first half from the N-terminus contains several repeat regions of the consensus sequence KTKEGV and is considered as the membrane interaction domain; the middle region, referred to as a non-amyloid-component region (NAC), is the region prone for aggregation; and the highly acidic C-terminus region maintains the protein in the solubilized form and functions as a chaperone [6], [39]. Consistent with this model, we found that unlike WT-syn, 112-syn loses chaperone-like activity as analyzed by the heat induced aldolase aggregation assay and this finding corroborates with earlier report [Fig. 1A and B][40]. Moreover, 112-syn alone exhibited an enhancement of absorbance in a dose dependent manner, which was further increased in presence of aldolase and this unique observation prompted us to study in detail its aggregation potential [Fig. 2].

The present study employing purified proteins of WT and 112-syn demonstrated that112-syn possess a temperature dependent aggregation propensity and this feature was lacking in WT-syn. In fact, a significant increase in 112-syn aggregation was evident at as low as 45°C within 10 min and the effects were observed to be dose-dependent. To our knowledge, the present study happens to be the first one to identify the temperature-dependent aggregation of 112-syn in a cell free assay system [Fig. 2 A and B].

Earlier reports demonstrated the tendency of WT-syn to form fibrillar structures at room temperatures following prolonged incubation [42]. However, the present study identified the enhanced fibrillation potential of 112-syn as compared to its wild-type counterpart and the fibrillar structures exhibited enhanced ThT fluorescence unlike the heat-induced aggregates. Though fibrillation of WT-syn was noticed following prolonged incubation corroborating with earlier findings (data not shown) [42], no gross fibrillation was evident during the initial 24 h time period [Fig. 4A]. These findings clearly indicate that 112-syn possesses accelerated potential towards fibrillation in addition to heat-induced aggregation. The inherent chaperone-like activity of WT-syn in preventing the non-native conformations of other proteins has been reported previously [39], [40]. Further, we also noticed that WT-syn dose-dependently inhibits the heat-induced aggregation of 112-syn [Fig. 3A and B]. Recently, it was reported that the C-terminus truncated form of α-syn induced the aggregation of WT-syn [25]. In the present study, we observed that WT-syn exacerbated fibrillation of 112-syn *in vitro* in a dose dependent manner [Fig 5A and B], however, the implications of the observed findings needs to be examined *in vivo*. Overall, the present results clearly indicate a possible cross-talk between α-syn isoforms in protein aggregation and provide us with new information on the possible biological consequences especially when the ratios of α-syn isoforms are altered. Moreover, our earlier study identified induced-alternative splicing of α-syn resulting in the generation of 112-syn by various Parkinsonian mimics leading to gross alterations in the ratios of WT-syn: 112-syn in cellular models of PD [35]. In addition, the existence of altered levels of α-syn isoforms in patients suffering from PD, DLB and synucleinopathies have been recently reported [36], [37]. In light of the above findings, the observed aggregation potential of 112-syn in conjunction with its inter-dependence on the relative abundance of WT-syn might bear serious biological consequences and underscores the importance of plausible cross-talk between α-syn isoforms in the pathophysiology of PD or related pathologies. However, further *in vivo* studies are warranted to pin-point the relative contribution of individual isoforms of α-syn in disease pathology.
